# A double-layer plate method for rapid screening of cell wall-targeting antibiotic producers using a microbial whole-cell biosensor

**DOI:** 10.1128/aem.01207-26

**Published:** 2026-07-21

**Authors:** Jianping Xu, Chiheng Gong, Jiaqi Yu, Yiwei Hu, Jingfeng Zhang, Jingxiao Cai, Qiu Meng, Zhiliang Yu, Jianhua Yin

**Affiliations:** 1 College of Biotechnology and Bioengineering, Zhejiang University of Technology 630365, Hangzhou, China; 2 State Key Laboratory of Green Chemical Synthesis and Conversion, Zhejiang University of Technology, Huzhou, China; Michigan State University, East Lansing, Michigan, USA

**Keywords:** whole-cell biosensor, cell wall-targeting antibiotics, double-layer plate method, antibiotic producer

## Abstract

The escalating antimicrobial resistance crisis demands innovative strategies for antibiotic discovery. Conventional approaches for identifying antibiotic-producing microorganisms from environmental samples are often laborious and low-throughput, requiring prior isolation and purification of individual strains. Here, we developed a high-throughput screening platform integrating a microbial whole-cell biosensor into a double-layer plate assay, enabling rapid identification of bacteria producing cell wall-targeting antibiotics from environmental samples. The biosensor is based on the PghKR two-component system from the gram-negative bacterium *Shewanella oneidensis* MR-1. Upon exposure to cell wall-targeting antibiotics, PghKR activates the promoter of *blaA*, driving expression of the *luxCDABE* reporter and generating luminescence. A highly sensitive biosensor was engineered through the synergistic deletion of *blaA* and *ampG*, which greatly improved its responsiveness. The method was then applied to screen soil samples. From the primary screen, 103 colonies producing distinct luminescent signals were identified. Of these, 36 isolates consistently activated the biosensor in a confirmation assay, and 5 exhibited antibacterial activity against a multidrug-resistant indicator strain. This integrated approach combines microbial separation with immediate biosensor-based detection, thereby accelerating the discovery of novel antibiotic producers from complex environmental communities.

**IMPORTANCE:**

The rise of antimicrobial resistance calls for faster, more efficient ways to discover new antibiotics from environmental microbes. Traditional methods are slow because they require laborious, one-by-one isolation and purification of individual strains before any activity testing. To overcome this bottleneck, we developed a simple double-layer plate assay that directly identifies bacteria producing cell wall-targeting antibiotics while they grow. This “grow-and-detect” strategy bypasses traditional isolation steps, dramatically speeding up the initial discovery pipeline. Our platform enables large-scale, low-cost screening of environmental samples for antibiotic producers.

## INTRODUCTION

The discovery of antibiotics stands as one of the most transformative advances in modern medicine. However, the rapid and widespread emergence of antimicrobial resistance (AMR) has escalated into a global health crisis. Without effective intervention, it is estimated that annual deaths directly attributable to AMR will reach 1.91 million, with an additional 8.22 million deaths associated with AMR (1, 2). This alarming trend underscores the urgent need for innovative and efficient strategies to discover novel antibacterial agents capable of overcoming resistant pathogens.

The identification of antibiotic-producing microorganisms is a critical initial step in the discovery of novel antimicrobial agents. Conventional phenotypic screening typically requires the prior isolation and purification of individual microbial strains, followed by antimicrobial activity and mode-of-action assays (3–8). This multi-step process is labor-intensive and low-throughput and fails to identify the molecular target of active compounds. In contrast, whole-cell biosensors directly couple antibiotic detection with a defined cellular response (9, 10). These biosensors utilize engineered microorganisms to convert the presence of a specific antibiotic, or the cellular stress it induces, into a quantifiable reporter signal such as fluorescence or luminescence (11).

To date, several whole-cell biosensors have been developed for specific antibiotic classes, including β-lactams (12–14), tetracyclines (15–17), macrolides (18–21), and amphotericin-like polyenes (22), as well as for key cellular processes such as cell envelope synthesis, DNA damage, and protein synthesis (23–28). Some of these biosensors have been successfully applied for high-throughput screening of compound libraries, such as a highly specific *Staphylococcus aureus* biosensor for cell wall biosynthesis inhibitors tested against natural product libraries (24, 25). Others have been adapted to double-layer plate assays for detecting antibiotic producers. Examples include a nisin-responsive biosensor to identify nisin-producing bacteria from food samples and a panel of *Bacillus subtilis* bioreporters for mode-of-action profiling of pure actinomycete strains (29, 30). However, these approaches still rely on prior isolation and purification of individual microbial strains rather than enabling direct screening of complex environmental samples (26–30)

The bacterial cell wall is essential for survival by maintaining cellular morphology and providing protection against osmotic stress (31). Peptidoglycan, the major component of the cell wall, represents a classic and highly selective target for antibiotics because its biosynthetic pathway is absent in mammalian cells. Several clinically important classes of antibiotics, including β-lactams (e.g., ampicillin), glycopeptides, fosfomycin, and D-cycloserine, exert their effects by inhibiting distinct steps of peptidoglycan biosynthesis (32–34). Recent advances have expanded the repertoire of potential targets through the identification of key enzymes such as the lipid II flippase MurJ and the SEDS (shape, elongation, division, and sporulation) family proteins (35–37). Moreover, newly discovered antibiotics such as corbomycin, complestatin, clovibactin, and Novltex disrupt peptidoglycan metabolism via previously unknown mechanisms (38–40). Together, these findings indicate that many unexplored antibiotics targeting cell wall biosynthesis remain to be discovered.

Recently, we identified a novel two-component system in *Shewanella oneidensis* MR-1, designated PghKR, which specifically senses and responds to peptidoglycan damage induced by various cell wall-targeting antibiotics (J. Yin, submitted for publication). In this system, the histidine kinase PghK detects cell wall stress and phosphorylates the response regulator PghR, which in turn activates the expression of several genes including the β-lactamase gene *blaA* (27, 41, 42). Using this regulatory circuit, we previously engineered a whole-cell biosensor by fusing the *blaA* promoter (P*_blaA_*) to a *luxCDABE* reporter (P*_blaA_-lux*) (27). In the presence of cell wall-targeting antibiotics, the PghKR system triggers the expression of *luxCDABE* driven by P*_blaA_*, resulting in real-time luminescence detection. Deletion of *blaA* or the permease gene *ampG* further improved biosensor performance (27). Unlike most reported cell wall-targeting biosensors constructed in gram-positive hosts (23, 24), this biosensor is based on the gram-negative bacterium *S. oneidensis* MR-1, making it more suitable for screening antibiotics that target gram-negative pathogens (27).

In this study, we further enhanced the sensitivity of the PghKR-derived biosensor through the simultaneous deletion of *blaA* and *ampG*. Using this optimized biosensor, we developed an integrated screening platform that directly couples the cultivation of environmental microorganisms with real-time detection. This system enables the rapid, high-throughput identification of bacteria that produce cell wall-targeting antibiotics directly from complex microbial communities.

## RESULTS

### A concept for rapid screening of cell wall-targeting antibiotic producers using the double-layer plate method

**Fig 1:**
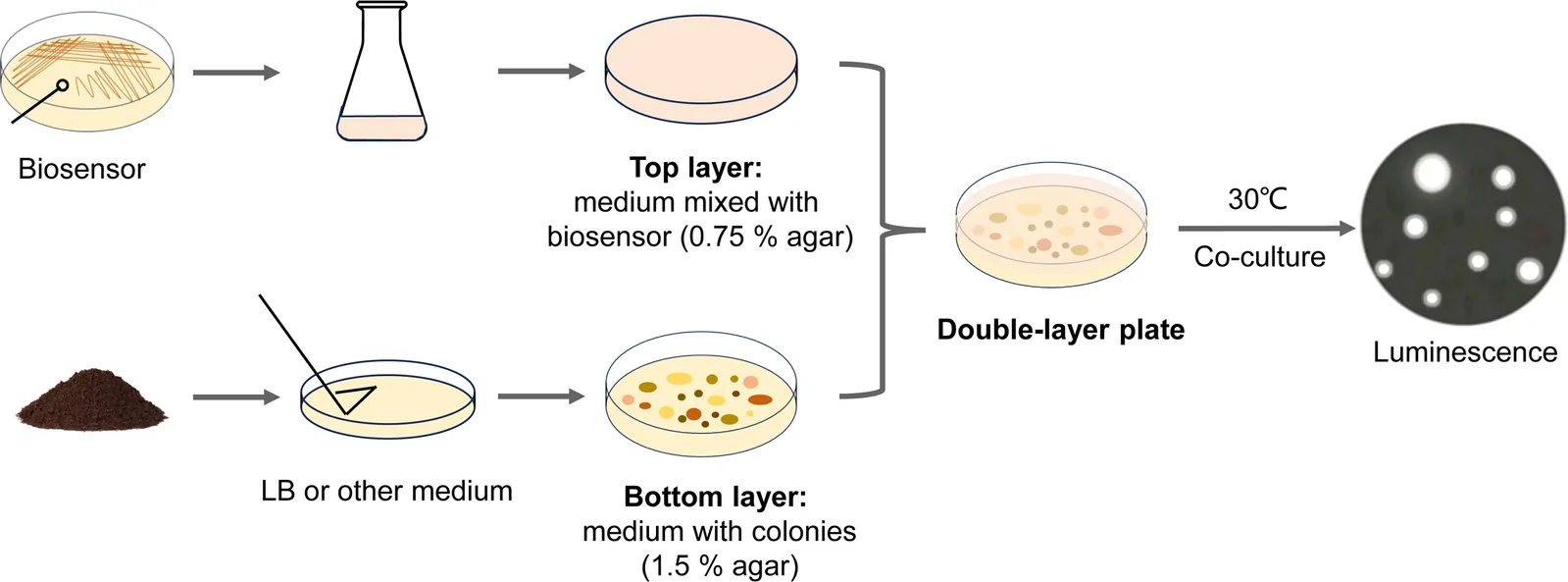
Schematic of the double-layer plate method for rapid screening of bacteria producing cell wall-targeting antibiotics directly from environmental samples.

Conventional methods for discovering antibiotic-producing bacteria from environmental samples are often laborious and time-consuming, as they typically require the isolation and purification of numerous microorganisms prior to functional testing. To address this limitation, we developed a novel screening strategy that directly couples initial microbial separation with immediate biosensor-based detection in a double-layer plate (Fig. 1).

The double-layer plate consists of two layers: a bottom layer of solid nutrient medium (e.g., Luria-Bertani [LB], 1.5% agar), which is inoculated with a serially diluted environmental sample to allow the formation and growth of discrete colonies, and a top layer of soft agar (e.g., Mueller-Hinton II [MH II], 0.75% agar) containing a microbial biosensor that detects cell wall-targeting antibiotics. After a pre-cultivation period to allow colony development on the bottom layer, the top layer is overlaid. As colonies grow, any cell wall-targeting antibiotics they produce diffuse into the top agar, activating the biosensor and generating a luminescent signal around producer colonies. This method allows immediate visual screening of antibiotic producers without the need for prior isolation.

### Construction of a high-sensitivity microbial biosensor for the detection of cell wall-targeting antibiotics

**Fig 2:**
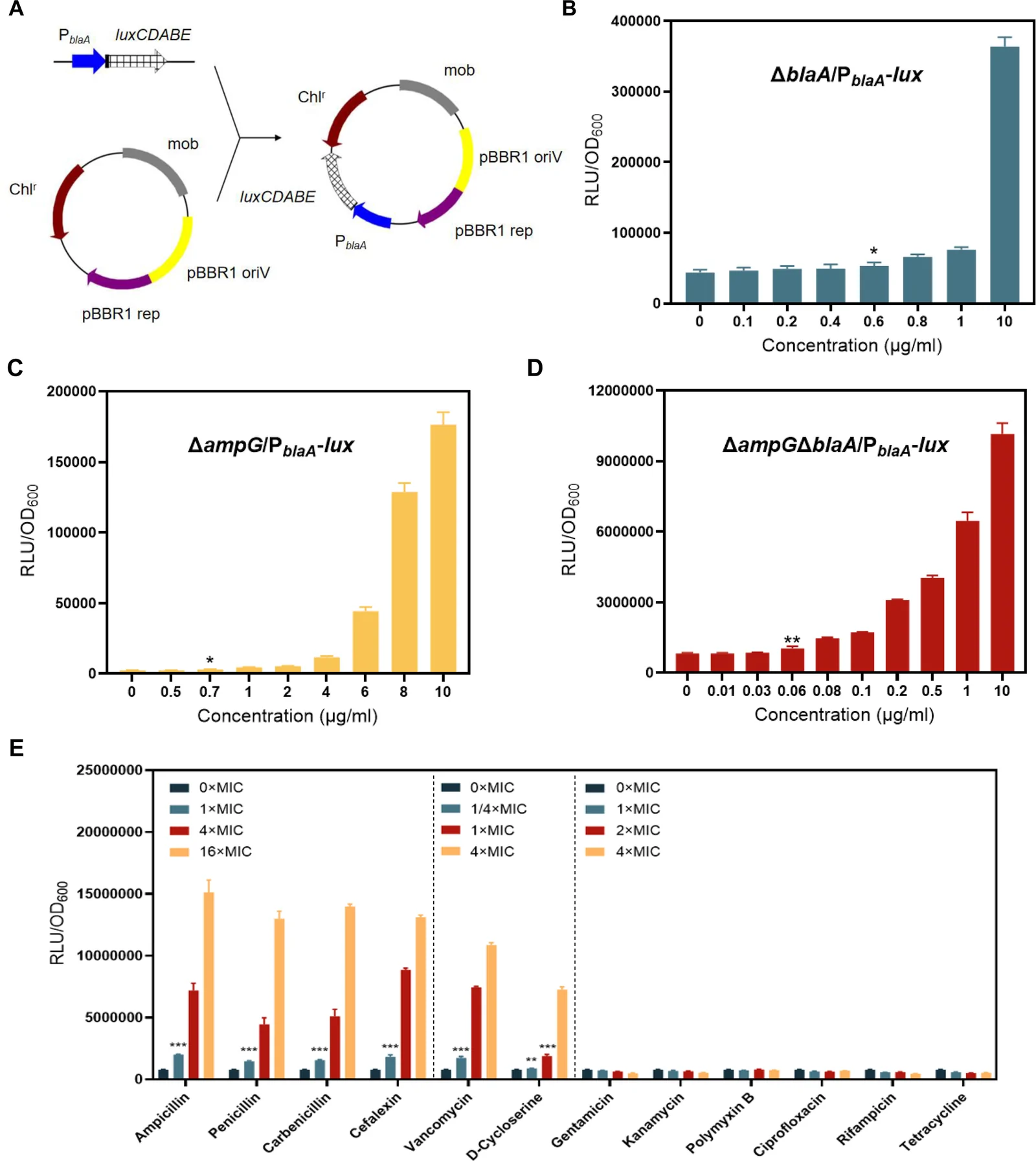
Construction and performance evaluation of the PghKR biosensors. (**A**) Schematic of the construction of reporter plasmid pBBR-P*_blaA_-lux*. (**B–D**) Dose-response of the three biosensors upon exposure to increasing concentrations of ampicillin: (**B**) Δ*blaA*/P*_blaA_-lux*, (**C**) Δ*ampG*/P*_blaA_-lux*, and (**D**) Δ*ampG*Δ*blaA*/P*_blaA_-lux*. The lowest ampicillin concentration yielding a statistically significant signal is labeled for each biosensor. (**E**) Specificity of the Δ*ampG*Δ*blaA*/P*_blaA_-lux* biosensor. The biosensor was tested against various antibiotics targeting the cell wall or other cellular processes. Antibiotics were tested at concentrations relative to their respective MICs, and the MIC values are provided in the Supplementary Material (Table S1). Significance levels: *, *P* < 0.05; **, *P* < 0.01; ***, *P* < 0.001.

Based on our previously developed biosensors Δ*blaA*/P*_blaA_-lux* and Δ*ampG*/P*_blaA_-lux* (27), we hypothesized that simultaneously deleting both *blaA* and *ampG* in *Shewanella oneidensis* MR-1 would synergistically enhance the detection sensitivity for cell wall-targeting antibiotics. To test this, a new biosensor was constructed by introducing the P*_blaA_-lux* reporter cassette into the Δ*ampG*Δ*blaA* strain, which was designated as *ampG*Δ*blaA*/P*_blaA_-lux* (Fig. 2A).

Using ampicillin as a representative cell wall-targeting antibiotic, we determined the detection limits of the three biosensors by measuring luminescence after 1 h of exposure to a range of concentrations (Fig. 2B through D). All three biosensors exhibited a concentration-dependent increase in luminescence. The Δ*blaA* and Δ*ampG* biosensors exhibited detection limits of approximately 0.6 and 0.7 μg/mL, respectively. The newly constructed Δ*ampG*Δ*blaA* biosensor showed a significantly lower detection limit of 0.06 μg/mL. These results confirm that the combined deletion of the two genes produces a synergistic effect that substantially enhances the sensitivity of the biosensor.

The specificity of the Δ*ampG*Δ*blaA*/P*_blaA_-lux* biosensor was next evaluated by testing a panel of antibiotics with different cellular targets (Fig. 2E). In response to various cell wall-targeting antibiotics, including β-lactams (three penicillins and cephalexin), the glycopeptide vancomycin, and the cyclic peptide D-cycloserine, the biosensor showed a concentration-dependent and markedly increased luminescence. In contrast, no significant increase in luminescence was detected upon exposure to antibiotics that target other cellular processes, such as protein synthesis (gentamicin, kanamycin, and tetracycline), membrane integrity (polymyxin B), DNA replication (ciprofloxacin), or RNA synthesis (rifampin) (Fig. 2E). These results demonstrate that the Δ*ampG*Δ*blaA*/P*_blaA_-lux* biosensor responds specifically to cell wall-targeting antibiotics.

### Detection of diverse cell wall-targeting antibiotics by the Δ*ampG*Δ*blaA* biosensor on solid medium

**Fig 3:**
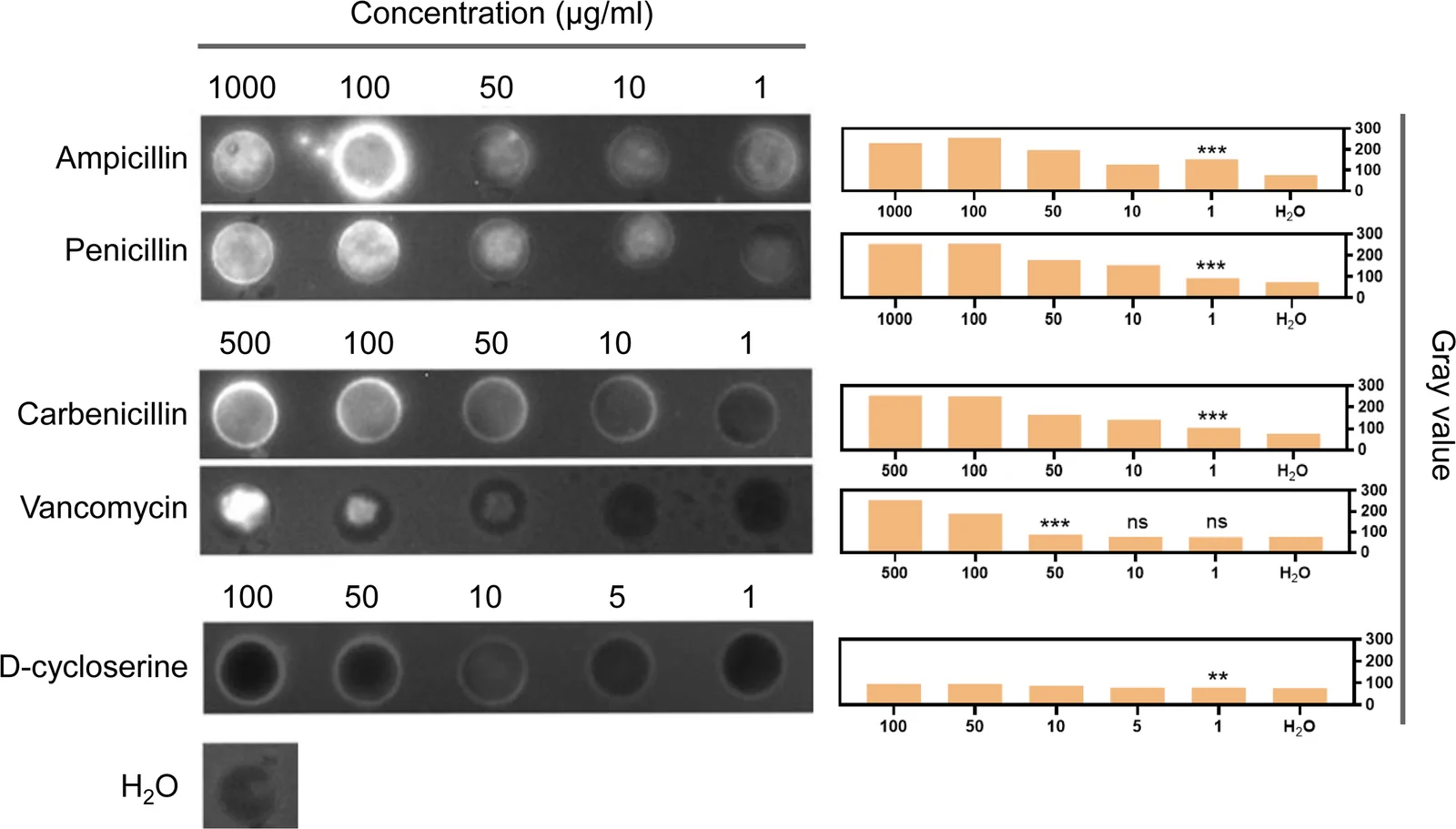
Detection of diverse cell wall-targeting antibiotics by the Δ*blaA*Δ*ampG* biosensor on solid medium. Left panels: luminescence images showing the response of the biosensor to various cell wall-targeting antibiotics (three β-lactams, vancomycin, and D-cycloserine) at increasing concentrations. H₂O was included as a negative control. Right panels: quantitative analysis of the left panel images using ImageJ, presented as gray value. The lowest antibiotic concentration that produced a statistically significant signal is indicated by significance markers. Significance levels: **, *P* < 0.01; ***, *P* < 0.001.

To assess the performance of the Δ*ampG*Δ*blaA*/P*_blaA_-lux* biosensor on solid medium, we tested its response to the same panel of cell wall-targeting antibiotics used in the liquid assays, including three β-lactams (ampicillin, penicillin, and carbenicillin), vancomycin, and D-cycloserine. As shown in Fig. 3, the biosensor exhibited concentration-dependent luminescence in response to all antibiotics tested. The three β-lactams produced strong and clear signals across a broad concentration range, with detectable luminescence at concentrations as low as 1 μg/mL. Vancomycin also triggered a notable response, yielding visible luminescence at 50 μg/mL. Although the response to D-cycloserine was weaker than those induced by the other antibiotics, it remained clearly detectable. These results confirm that the Δ*ampG*Δ*blaA* biosensor is functional on solid medium and capable of detecting a broad spectrum of cell wall-targeting antibiotics.

### Optimization of the double-layer plate screening method

**Fig 4:**
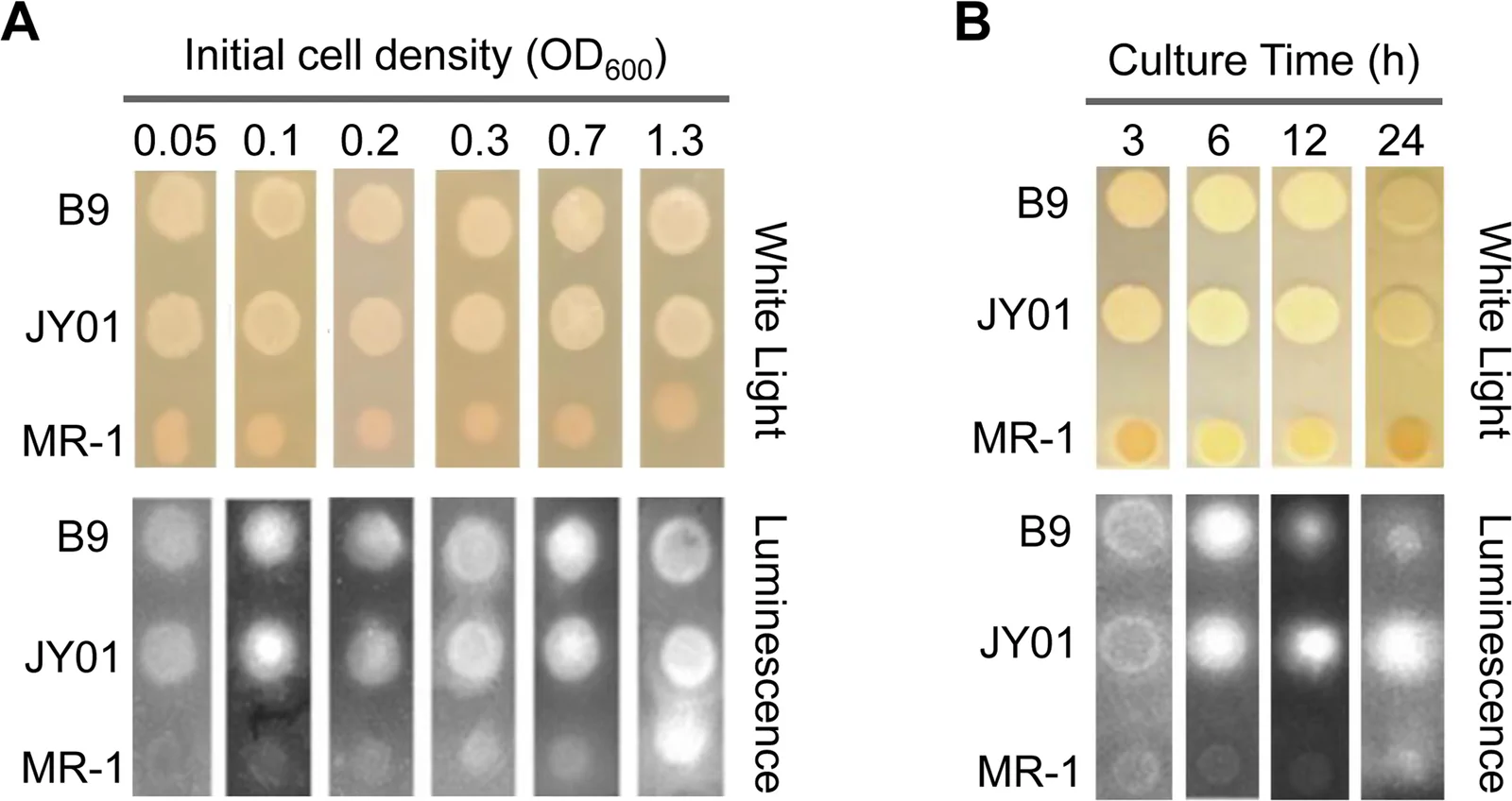
Optimization of the double-layer plate screening method. (**A**) Optimization of the initial cell density (OD_600_) of the biosensor. (**B**) Optimization of the co-cultivation time. In both experiments, signals from two antibiotic producers (*Bacillus* sp. B9 and JY01) and a non-producer control (*S. oneidensis* MR-1) were compared, arranged from top to bottom on each plate in the order: *Bacillus* sp. B9, JY01, and MR-1. For each plate, white light images and corresponding luminescence images are shown. Luminescence images were captured with an exposure time of 1 min.

The performance of the double-layer plate screening method is governed by two key parameters, the initial cell density of the biosensor strain and the subsequent co-cultivation time on the plate. A lower initial cell density increases the susceptibility of population to antibiotic stress, thereby amplifying the P*_blaA_*-mediated luminescent response. Concurrently, the cultivation time must be long enough to permit antibiotic diffusion, promoter induction, and signal accumulation, while sufficiently short to prevent biosensor overgrowth or stress-induced false positives. We therefore optimized both parameters (Fig. 4).

To determine the optimal initial cell density, the biosensor strain was applied to the top agar layer at optical density at 600 nm (OD_600_) values ranging from 0.05 to 1.3. The assay was performed on double-layer plates containing two antibiotic producers (*Bacillus* sp. B9 and JY01) and a non-producer (*S. oneidensis* MR-1). The strongest luminescent signals from the producers were observed at an OD_600_ of 0.1, while weaker responses were observed at other cell densities tested (0.05, 0.3, 0.5, 0.7, and 1.3) (Fig. 4A). The non-producer MR-1 also exhibited the weakest background luminescence at an OD_600_ of 0.1, resulting in the best signal-to-noise ratio. Notably, at the highest cell density (OD_600_ of 1.3), MR-1 itself generated a strong signal, indicating a high risk of false positives (Fig. 4A). Therefore, an OD_600_ of 0.1 was selected as the optimal initial cell density for subsequent assays.

We next determined the optimal co-cultivation time by monitoring luminescence development over 24 h (Fig. 4B). The non-producer MR-1 exhibited only weak background luminescence throughout the experiment. In contrast, both *Bacillus* sp. B9 and JY01 induced strong signals, which became clearly detectable after 6 and 12 h of incubation, respectively (Fig. 4B). However, the luminescence intensity of *Bacillus* sp. B9 began to decrease after 12 h. A co-cultivation time of 6 h was therefore selected as optimal, as it provided a strong and specific signal while avoiding signal decay.

### Screening of putative cell wall-targeting antibiotic producers from soil

**Fig 5:**
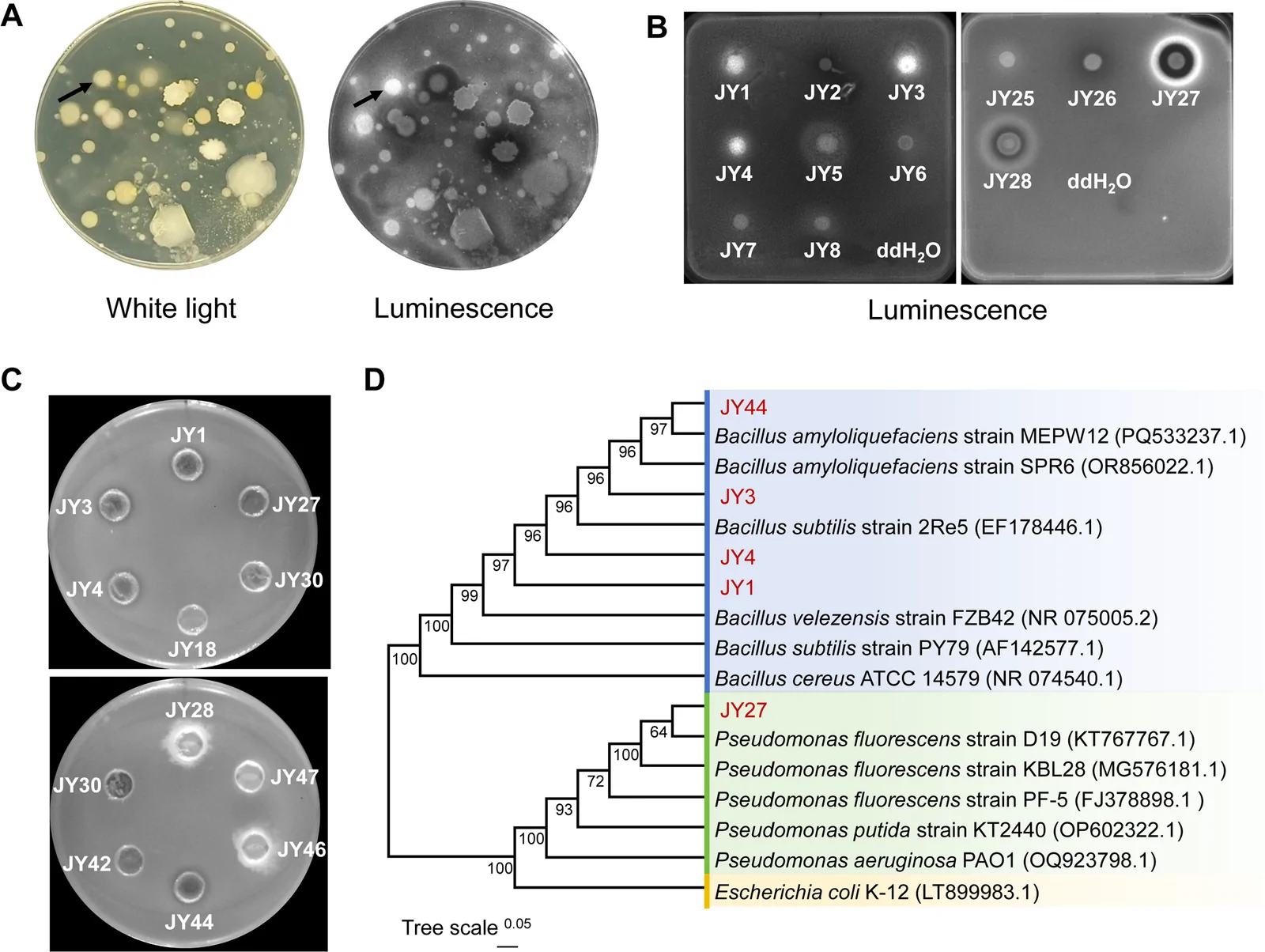
Screening and validation of cell wall-targeting antibiotic producers from soil. (**A**) Representative primary screening plate. The left panel shows the white light image, and the right panel shows the corresponding luminescence image. Arrows indicate colonies that induced distinct luminescent signals, identifying them as candidate antibiotic producers. (**B**) Confirmation assay with purified isolates. Luminescence images of representative candidate isolates are shown. Isolates such as JY1, JY3, JY4, JY5, JY27, and JY28 consistently activated the biosensor (positives), whereas other isolates were verified as false positives. ddH₂O served as a negative control. The validation results for the remaining positive isolates are provided in Fig. S1. (**C**) Antibacterial activity of the five confirmed active isolates (JY1, JY3, JY4, JY27, and JY44) against the multidrug-resistant indicator strain *Pseudomonas* sp. BR8. Visible inhibition zones are indicated. Several other positive isolates (e.g., JY18, JY28, JY30, JY42, JY46, and JY47) did not produce inhibition zones against BR8. (**D**) Phylogenetic tree of the five active isolates (JY1, JY3, JY4, JY27, and JY44) based on 16S rRNA gene sequences. Sequences were aligned using MEGA 11, and a bootstrap consensus tree (1,000 replicates) was constructed with the neighbor-joining method. The tree was visualized and optimized using iTOL. Colony morphologies of these five isolates are shown in Fig. S3.

To validate the application of the optimized double-layer plate method, we performed a large-scale screening of soil samples collected from multiple regions in China. Serial dilutions of soil suspensions were spread onto the surface of the bottom agar and incubated until visible colonies formed. The Δ*ampG*Δ*blaA* biosensor was suspended in soft agar and then overlaid as the top layer. After 6 h of further incubation, colonies were examined for luminescence. A colony was scored as positive if it produced a clearly discernible luminescent signal that was visibly brighter than the background and surrounding colonies without detectable luminescence (Fig. 5A). To avoid repeatedly isolating the same strain, only one representative colony per morphotype (based on shape, color, texture, size, etc.) was selected. Using these criteria, a total of 103 colonies that induced distinct luminescent signals were initially identified. All candidate colonies were carefully purified and subjected to a confirmation assay under the same double-layer conditions. Of these, 36 isolates consistently activated the biosensor (Fig. 5B; Fig. S1), confirming their potential to produce diffusible compounds that induce the cell wall stress response. This result demonstrates the reliability and effectiveness of the method for primary screening of antibiotic producers.

To further validate this method, the antibacterial activity of the 36 isolates was assessed using *Pseudomonas* sp. BR8 as an indicator strain. This strain, which was previously characterized in our laboratory, exhibits multidrug resistance to various cell wall-targeting antibiotics (including penicillin, vancomycin, D-cycloserine, cephalexin, cephalosporin C, bacitracin, and fosfomycin) (Fig. S2). Results showed that five isolates (JY1, JY3, JY4, JY27, and JY44) produced clearly visible inhibition zones against BR8 (Fig. 5C). Based on 16S rRNA gene sequencing and BLAST analysis, four isolates (JY1, JY3, JY4, and JY44) were identified as members of the genus *Bacillus*, while the remaining strain (JY27) was identified as *Pseudomonas* sp. (Fig. 5D; Fig. S3).

## DISCUSSION

The double-layer plate method developed in this study integrates a microbial whole-cell biosensor into a “grow-and-detect” workflow, enabling rapid and direct identification of bacteria producing cell wall-targeting antibiotics from complex soil samples. By coupling microbial cultivation with real-time functional detection, our approach bypasses the labor-intensive steps of prior isolation and purification, thereby significantly accelerating the primary screening process (Fig. 1).

Several whole-cell biosensor-based strategies have been developed for antibiotic discovery. One common approach is to screen compound libraries (e.g., natural product or synthetic collections) for antibacterial activity, often using biosensors that target specific pathways (22–25). Another strategy employs double-layer plate assays to identify antibiotic-producing microorganisms directly from cultivated samples (26–28, 30). However, in most cases, these methods still require prior isolation and purification of individual microbial strains. Notably, one study achieved direct screening of nisin-producing bacteria from the simplified matrix of raw milk using a bioluminescence-based biosensor overlay method (29). In contrast, our double-layer plate method enables direct screening of antibiotic producers from complex environmental samples (e.g., soil) without prior isolation.

Notably, most reported cell wall-targeting biosensors are constructed in gram-positive hosts (e.g., *Bacillus subtilis* or *Staphylococcus aureus*) (23–25, 28). We previously developed a whole-cell biosensor for cell wall-targeting antibiotics based on the PghKR two-component system in the gram-negative bacterium *S. oneidensis* MR-1 (27). Deletion of *blaA* or *ampG* individually enhances biosensor sensitivity through distinct mechanisms. *blaA* deletion prevents antibiotic degradation, whereas *ampG* deletion blocks peptidoglycan recycling, leading to accumulation of cell wall damage signals in the periplasm and thereby facilitating PghKR activation (27, 42–44). Building on these findings, our double-deletion (Δ*blaA*Δ*ampG*) biosensor achieves a 10-fold lower detection limit for ampicillin (0.06 μg/mL) compared to single deletion biosensors, while retaining strict specificity for cell wall-targeting antibiotics (Fig. 2E). Importantly, given that the World Health Organization priority pathogens list is dominated by gram-negative bacteria (45), our gram-negative host-based biosensor provides a more physiologically relevant platform for screening antibiotics against these pathogens.

Despite its high sensitivity and specificity, we acknowledge an inherent limitation of *lux*-based whole-cell biosensors. The luminescence output can be influenced not only by specific promoter induction but also by the intracellular redox homeostasis of the biosensor cells (46, 47). As demonstrated by Nikel et al. (46), compounds that alter the cellular NADPH/NADP^+^ ratio may either enhance or suppress light emission, potentially generating false-positive signals. This phenomenon likely explains our observation that the non-producer *S. oneidensis* MR-1 generated a strong luminescent signal at the highest initial cell density tested (OD_600_ of 1.3) (Fig. 4), where the biosensor cells experienced metabolic stress and possible redox imbalance. Therefore, careful optimization of initial cell density and co-cultivation time, as performed in this study, is essential to minimize redox-related interference.

A central challenge in natural product discovery is dereplication, that is, the rediscovery of known antibiotics. Although computational approaches, including artificial intelligence, are creating new opportunities to identify novel chemical scaffolds (48–50), our work offers a complementary experimental strategy. The primary biosensor-based screen, followed by an antibacterial assay using multidrug-resistant indicator strains (Fig. 5C), not only prioritizes isolates with activity against resistant pathogens but also helps to exclude those producing antibiotics with already characterized mechanisms.

Furthermore, the double-layer plate method can be readily adapted to screen for inhibitors of other essential pathways, as a wide range of whole-cell biosensors have been engineered to detect various antibiotic classes (11–28). Future work could develop biosensors capable of detecting two or more cellular targets simultaneously, which would facilitate the identification of agents acting on multiple pathways and thereby help mitigate the emergence of resistance.

In summary, the double-layer plate method serves as an efficient, high-throughput platform for the primary enrichment of microbial candidates with a high potential to produce bioactive molecules. Chemical elucidation and mechanistic investigation of the active compounds produced by these candidates will be the focus of future work.

## MATERIALS AND METHODS

### Bacterial strains, plasmids, and growth conditions

All strains used in this study are listed in Table 1. *Escherichia coli* WM3064 strain was a 2,6-diaminopimelic acid (DAP) auxotroph and used as the donor for conjugation. *E. coli* strains were grown in Luria-Bertani (LB) medium at 37°C, while *S. oneidensis* and *Pseudomonas* strains were cultured in LB medium at 30°C. When required, media were supplemented with 5.7 μg/mL 2,6-diaminopimelic acid (DAP) and/or 25 μg/mL chloramphenicol.

**TABLE 1:** Strains and plasmids used in this study

Strain or plasmid	Description	Source
Strains		
*E. coli*		
DH5α	Host strain for plasmids	Lab stock
WM3064	Donor strain for conjugation; Δ*dapA*	Metcalf, UIUC
WM3064/P*_blaA_-lux*	WM3064 harboring plasmid P*_blaA_-lux*	(27)
*S. oneidensis*		
MR-1	Wild type	Lab stock
Δ*blaA*	*blaA* deletion strain	(44)
Δ*ampG*	*ampG* deletion strain	(44)
Δ*ampG*Δ*blaA*	*ampG* and *blaA* deletion strain	This study
Δ*blaA*/P*_blaA_-lux*	Δ*blaA* harboring plasmid pBBR-P*_blaA_-lux*	(27)
Δ*ampG*/P*_blaA_-lux*	Δ*ampG* harboring plasmid pBBR-P*_blaA_-lux*	(27)
Δ*ampG*Δ*blaA*/P*_blaA_-lux*	Δ*ampG*Δ*blaA* harboring plasmid pBBR-P*_blaA_-lux*	This study
*Pseudomonas* sp. BR8	Multi-drug resistant bacteria	Lab stock
Plasmids		
pBBR-*lux*	Cm^r^; containing the luciferase gene *luxCDABE*	(27)
pBBR-P*_blaA_-lux*	Expression of *luxCDABE* controlled by the P*_blaA_* promoter	(27)

### Construction of the biosensor

The recombinant plasmid pBBR-P*_blaA_-lux* harboring *luxCDABE* was constructed previously (27). In this study, the plasmid was transferred into *S. oneidensis* Δ*ampG*Δ*blaA* via conjugation. Transconjugants were selected on LB agar containing chloramphenicol and verified by colony PCR, yielding the biosensor strain Δ*ampG*Δ*blaA*/P*_blaA_-lux*.

### Determination of relative luminescence units

Single colonies of Δ*blaA*/P*_blaA_-lux*, Δ*ampG*/P*_blaA_-lux*, and Δ*ampG*Δ*blaA*/P*_blaA_-lux* were inoculated into LB broth and grown overnight. Each culture was diluted 1:100 into 50 mL fresh LB medium and shaken at 30°C until OD_600_ (optical density at 600 nm) reached 0.1. For determination of the detection limits, 3 mL aliquots of each culture were treated with ampicillin at the final concentrations indicated in Fig. 2. For specificity evaluation, 3 mL aliquots of the Δ*ampG*Δ*blaA*/P*_blaA_-lux* culture were treated with a panel of antibiotics (ampicillin, penicillin, carbenicillin, cefalexin, vancomycin, D-cycloserine, gentamycin, kanamycin, polymyxin B, ciprofloxacin, rifampicin, and tetracycline) at concentrations relative to their respective MICs (see Table S1). After 1 h of incubation at 30°C with shaking, 200 μL of each sample was transferred to a white, clear-bottom 96-well microplate. Luminescence and OD_600_ were measured using a SynergyH1 microplate reader (BioTek, Winooski, VT). The relative luminescence unit normalized to cell density (RLU/OD_600_) was calculated as follows:


$$\frac{RLU}{OD_{600}}=\frac{RLU_{biosensor}-RLU_{LB}}{OD_{biosensor}-OD_{LB}}$$

### Validation of biosensor performance on solid medium

The Δ*ampG*Δ*blaA*/P*_blaA_-lux* biosensor was grown overnight and inoculated into 50 mL of fresh LB medium. After shaking at 30°C until OD_600_  =  0.1, the culture was mixed with molten MH II soft agar (0.75% agar) at a 1:5 (vol/vol) ratio and overlaid onto LB plates to form double-layer plates. Following solidification, filter-paper discs were placed on the plates, and 5 μL of each cell wall-targeting antibiotic (at concentrations shown in Fig. 3) was dropped onto each disc. Plates were incubated at 30°C for 1 h, after which luminescence was captured using an iBright 1500 imaging system (Thermo Fisher Scientific). For quantitative analysis, the gray value of each luminescence halo was measured using ImageJ software.

### Optimization of the double-layer plate assay

Two key parameters were optimized, including the initial cell density of the biosensor and the co-cultivation time. For cell density optimization, the Δ*ampG*Δ*blaA*/P*_blaA_-lux* biosensor was grown to OD_600_ values ranging from 0.05 to 1.3. At each OD_600_, cells were mixed with soft MH II agar (1:5, vol/vol) and overlaid onto LB plates pre-spotted with two antibiotic producers (*Bacillus* sp. B9 and *Bacillus* sp. JY01) and a non-producer (*S. oneidensis* MR-1). After 12 h of incubation, luminescence was recorded using a fixed exposure time of 1 min. For co-cultivation time optimization, double-layer plates prepared as above were incubated at 30°C for 3, 6, 12, and 24 h, after which luminescence was imaged under the same fixed exposure time.

### Screening for potential cell wall-targeting antibiotic producers from soil

Soil samples from multiple regions in China (Ningbo, Deqing, Baoding, Yantai, and Yuyao) were suspended in sterile water, vortexed, and serially diluted to 10^−3^. Aliquots (100 μL) were spread onto LB plates and incubated at 30°C for 24 h. A top layer of soft agar containing the Δ*ampG*Δ*blaA*/P*_blaA_-lux* biosensor was then overlaid. After 6 h of incubation at 30°C, luminescent signals were detected using the imaging system. Colonies that produced a luminescent signal visibly brighter than the background were selected as candidates. To avoid duplicate isolation of the same strain, only one representative colony per distinct morphotype (based on size, color, texture, etc.) was picked. Candidate colonies were purified by streaking, and each isolate was spotted onto fresh LB plates for confirmation under the same double-layer conditions.

### Antibacterial activity assay

The antibacterial activity of isolates was evaluated using an agar diffusion assay as previously described (51). The multidrug-resistant strain *Pseudomonas* sp. BR8 served as the indicator. This strain exhibits resistance to multiple cell wall-targeting antibiotics, including penicillin, vancomycin, D-cycloserine, cephalexin, cephalosporin C, bacitracin, and fosfomycin (Fig. S2). After pre-diffusion at 4°C for 1.5 h, plates were incubated at 30°C for 24 h, and inhibition zones were recorded.

### Molecular identification of candidate antibiotic-producing bacteria

16S rRNA gene fragments were amplified by PCR using universal bacterial primers (27-F: AGAGTTTGATCCTGGCTCAG/1492R: TACGGCTACCTTGTT ACGACTT). Purified PCR products were commercially sequenced, and the resulting sequences were subjected to BLAST analysis against the NCBI nucleotide database to determine the closest taxonomic relatives. For phylogenetic analysis, sequences were aligned with reference sequences using ClustalW in MEGA 11 software. A phylogenetic tree was constructed using the neighbor-joining method with 1,000 bootstrap replicates. The tree was visualized and optimized using iTOL.

### Statistical analyses

All experiments were performed in biological triplicates. Data are presented as mean ± standard deviations. Differences between groups were assessed using Student’s *t*-test, with significance levels denoted as the following: ns, not significant; *, *P*  <  0.05; **, *P*  <  0.01; *P* < 0.001.

## SUPPLEMENTAL MATERIAL

Supplemental material aem.01207-26-s0001.pdf Table S1; Fig. S1 to S3.

ASM does not own the copyrights to Supplemental Material that may be linked to, or accessed through, an article. The authors have granted ASM a non-exclusive, world-wide license to publish the Supplemental Material files. Please contact the corresponding author directly for reuse.
