# Supplementary material for: A double-layer plate method for rapid screening of cell wall-targeting antibiotic producers using a microbial whole-cell biosensor
Source: Appl Environ Microbiol. 2026 Jul 21;92(8):e01207-26. doi: 10.1128/aem.01207-26 (PMC13488354; doi:10.1128/aem.01207-26)
Supplement: Supplementary file 1 [file aem.01207-26-s0001.pdf]

Supplementary information for

A double-layer plate method for rapid screening of cell wall-targeting antibiotic producers using a microbial whole-cell biosensor

Jianping Xu[1], Chiheng Gong[1], Jiaqi Yu[1], Yiwei Hu[1], Jingfeng Zhang[1], Jingxiao Cai[1], Qiu Meng[1], Zhiliang Yu[1], Jianhua Yin[1,2]*

1. College of Biotechnology and Bioengineering, Zhejiang University of Technology, Hangzhou 310014, China
2. State Key Laboratory of Green Chemical Synthesis and Conversion, Zhejiang University of Technology, Huzhou 313099, China

Address correspondence to Jianhua Yin, jianhuay@zjut.edu.cn

Table S1 MICs of different antibiotics against the Δ*ampG*Δ*blaA* strain

Antibiotic name	Abbreviation	Antibiotic class	MIC(μg/mL)
Ampicillin	AMP	Penicillins	0.25
Penicillin G	PEN	Penicillins	0.5
Carbenicillin	CAR	Penicillins	2
Cefalexin	CFX	Cephalosporins	4
Vancomycin	VAN	Glycopeptides	64
D-cycloserine	DCS	Cycloserines	8
Gentamicin	GEN	Aminoglycosides	2
Kanamycin	KAN	Aminoglycosides	4
Polymyxin B	PMB	Polypeptides	0.125
Ciprofloxacin	CIP	Quinolones	0.0625
Rifampicin	RIF	Rifamycins	0.25
Tetracycline	TET	Tetracyclines	1

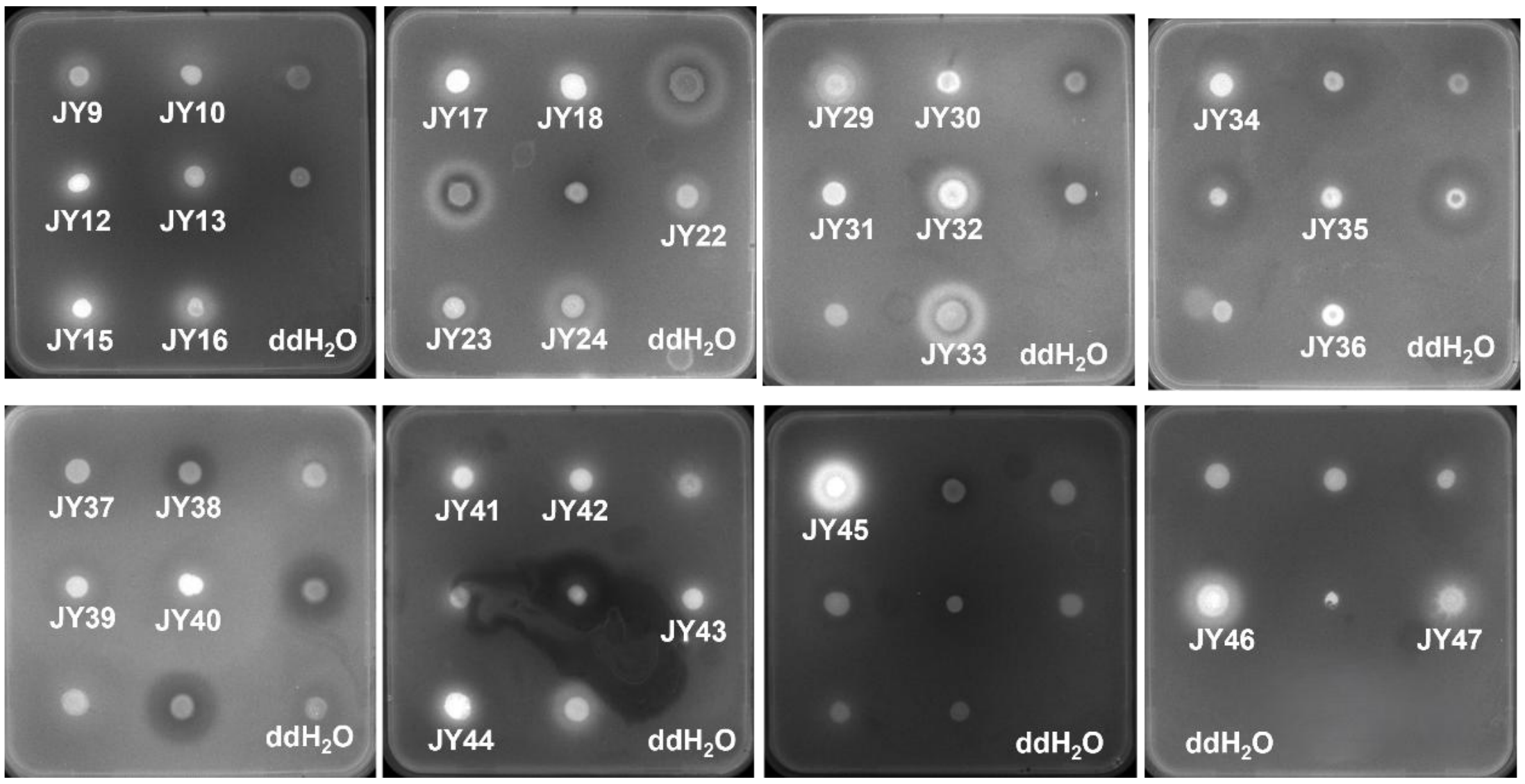

FIG S1 Confirmation assay of additional positive isolates (related to Fig. 5B). The luminescence images show the remaining positive isolates from the total of 36 that consistently activated the biosensor under the same double-layer conditions described in Fig. 5B. Only isolates that produced a clearly discernible luminescent signal are labelled. Each plate contains eight purified candidate isolates and a ddH_2O control.

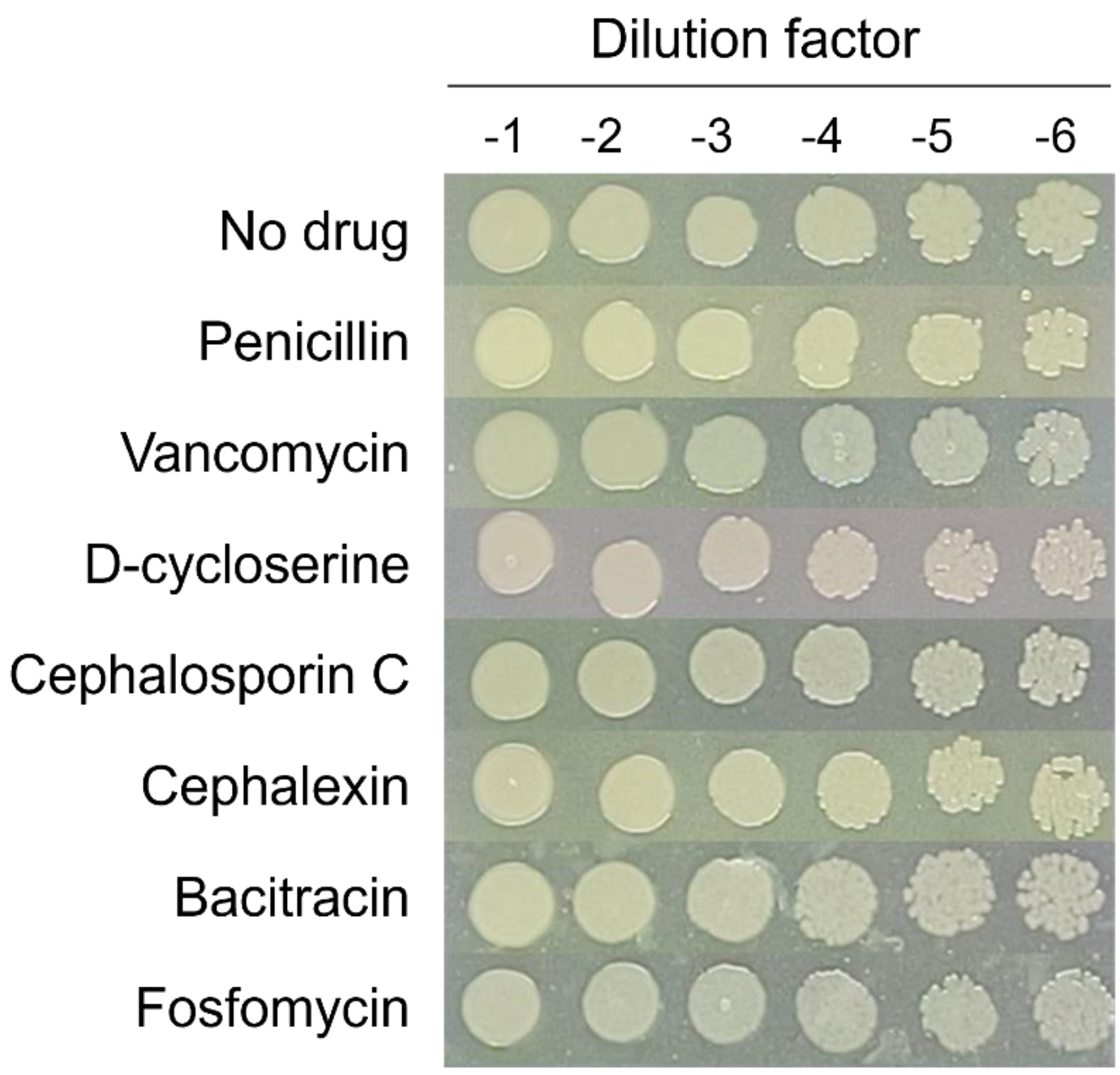

FIG S2 Susceptibility of *Pseudomonas* sp. BR8 to various cell wall-targeting antibiotics. Overnight cultures of BR8 were diluted 1:100 into fresh MH II broth and incubated at 30 °C until the OD_{600} reached approximately 0.4. Ten-fold serial dilutions of this culture were prepared, and 3 µL of each dilution was spotted onto MH II agar plates containing the indicated antibiotics at a concentration of 100 µg/mL. Plates without antibiotic served as a growth control. Plates were incubated at 30 °C for 24 h, and bacterial growth was then recorded.

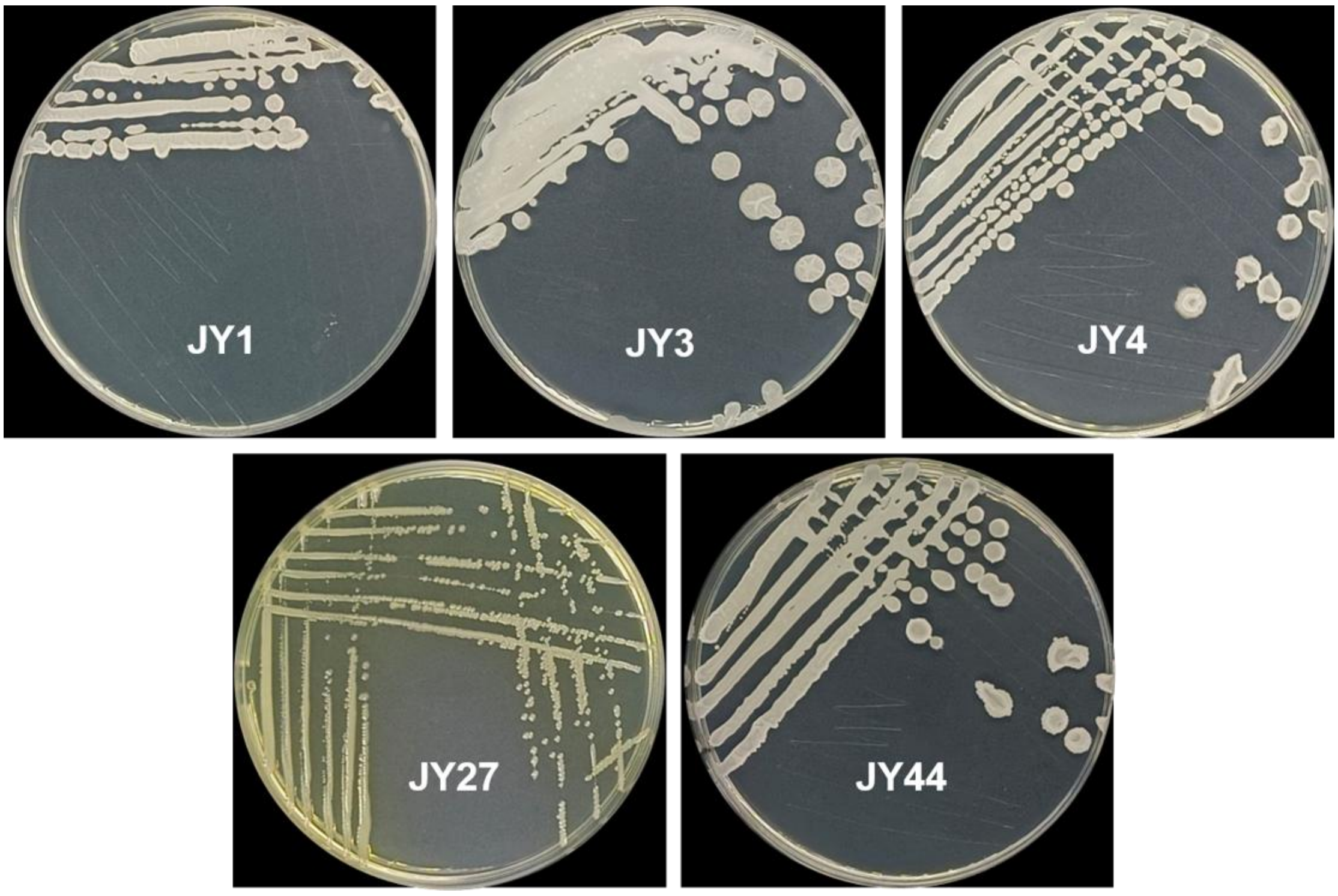

FIG S3 Colony morphology of the five active isolates (JY1, JY3, JY4, JY27, JY44) on LB agar. Each isolate was streaked and incubated at 30 °C for 24 h. These isolates correspond to the five strains shown in the phylogenetic tree (Fig. 5D) that showed antibacterial activity against *Pseudomonas* sp. BR8.
